# Combined Interval Cytoreductive Surgery and Carboplatin-Based Hyperthermic Intraperitoneal Chemotherapy in Advanced Primary High-Grade Serous Ovarian Cancer

**DOI:** 10.3390/curroncol30120748

**Published:** 2023-12-01

**Authors:** Claudèle Brault, Alexandre Brind’Amour, Lara de Guerke, Marie-Hélène Auclair, Lucas Sideris, Pierre Dubé, Mikaël Soucisse, Jean-François Tremblay, Laurence Bernard, Sabrina Piedimonte, Suzanne Fortin

**Affiliations:** 1 Department of Obstetrics and Gynecology, University of Montreal, Montreal, QC H3T 1J4, Canada; 2 Department of Surgery, CHU de Québec-Université Laval, Quebec, QC G1V 4G2, Canada; 3 Division of Surgical Oncology, Maisonneuve-Rosemont Hospital, Montreal, QC H1T 2M4, Canada; 4 Division of Gynecologic Oncology, Maisonneuve-Rosemont Hospital, Montreal, QC H1T 2M4, Canada; 5 Division of Gynecologic Oncology, Royal Victoria Hospital, Montreal, QC H4A 3J1, Canada

**Keywords:** HIPEC, carboplatin, ovarian cancer, interval cytoreductive surgery

## Abstract

Combining interval cytoreductive surgery (CRS) with hyperthermic intraperitoneal chemotherapy (HIPEC) improves survival in advanced epithelial ovarian carcinoma (EOC). Although limited, growing evidence regarding carboplatin-based HIPEC highlights its potential. This retrospective study included all patients with advanced primary high-grade serous ovarian cancer who underwent interval CRS combined with carboplatin-based HIPEC at our Canadian tertiary care center between 2014 and 2020. We identified 40 patients with a median age of 61 years. The median peritoneal cancer index was 13 and complete cytoreduction was achieved in 38 patients (95%). Median hospital stay was 13 days and there were four admissions to the intensive care unit (10%) and six readmissions (15%). Severe adverse events occurred in eight patients (20%) and there was no perioperative death. Recurrence was seen in 33 patients (82%) with a median DFS of 18.0 months and a median overall survival of 36.4 months. Multivariate analyses showed that age, peritoneal cancer index, completeness of cytoreduction, occurrence of severe complications, and bowel resection did not significantly impact DFS or OS in our cohort. Interval CRS combined with carboplatin-based HIPEC for advanced primary EOC is associated with acceptable morbidity and oncological outcomes. Larger studies are required to determine the long-term outcomes.

## 1. Introduction

Ovarian cancer is the fifth leading cause of cancer-related deaths in the United States, accounting for almost 14,000 deaths yearly [1]. Most patients present with advanced disease, with concomitant peritoneal metastases (PM) in up to 75% of cases at diagnosis [2]. The gold-standard treatment for patients with epithelial ovarian carcinoma (EOC) presenting with PM is the combination of cytoreductive surgery (CRS) and intravenous chemotherapy [3]. Completeness of cytoreduction (CC) [4], or amount of residual disease, is the strongest predictor of overall survival (OS). However, most patients will experience a recurrence, which leads to additional surgical and oncological challenges [5].

In the last 20 years, new approaches have been proposed to improve outcomes of patients with advanced primary EOC. Hyperthermic intraperitoneal chemotherapy (HIPEC) has gained interest after showing promising results [6], although it is not yet universally accepted. The rationale is to minimize microscopic residual peritoneal disease after CRS to prevent recurrence in the peritoneal cavity by directly exposing remaining tumoral implants to chemotherapy at an increased temperature (42 °C).

OVHIPEC-1, a randomized controlled trial published in 2018, demonstrated the survival benefit of combining interval CRS and cisplatin-based HIPEC in advanced EOC. The median OS was increased by almost 12 months, from 33.9 months in the surgery-only group to 45.7 months in the surgery with HIPEC group. Disease-free survival (DFS) was also improved without any significant increase in severe adverse events [7]. The updated final survival analysis at 10 years of the OVHIPEC-1 trial [8] reinforced the added benefits of HIPEC as this group had an improved survival, which was not related to the number of subsequent treatments. Based on these results, the National Comprehensive Cancer Network (NCCN) now recommends considering cisplatin-based HIPEC at the time of interval CRS for FIGO stage III disease in patients with stable or improving disease after at least three cycles of neoadjuvant chemotherapy (NACT) [9]. Its use remains at the physician’s discretion as an adjunct in these specific circumstances.

When cisplatin-based HIPEC is utilized, it is the mainstay of treatment in most HIPEC protocols for EOC. Data remain limited and contradictory regarding the use of carboplatin-based HIPEC to treat ovarian cancer. Earlier data suggested that carboplatin had less tumor penetrance and lacked synergistic benefits with hyperthermia for intraperitoneal use [10]. Longer exposure is needed to maximize the formation of its active metabolites [10]. Meanwhile, CRS techniques have become more extensive while potentially affecting carboplatin pharmacokinetics [11], mandating its reconsideration due to its favorable pharmacokinetic profile compared to cisplatin in systemic use [12]. At the molecular level, a new study of transcriptomic profiles of normal and ovarian cancer cells has demonstrated the upregulation of heat-shock-related genes driven by carboplatin-based HIPEC [13]. This process leads to the activation of an additional immune response, which further induces gene expression changes, providing a potential biological basis for its mechanism of action. These findings led to a new wave of publications and resuscitated the question of a potential alternative regimen. At least four prospective studies [11,14,15,16] and two recent phase II trials [17,18] have reported acceptable morbidity and shown promising oncological outcomes. Herein, we aim to evaluate the morbidity and oncological outcomes related to carboplatin-based HIPEC use during interval CRS.

## 2. Materials and Methods

This is a retrospective cohort study of 40 consecutive patients with primary advanced high-grade serous ovarian cancer and concomitant PM who underwent combined interval CRS and carboplatin-based HIPEC between July 2014 and March 2020 at an academic tertiary care center that specializes in the surgical management of peritoneal surface malignancies. This study was approved by the CIUSSS de l’Est-de-l’Île-de-Montréal ethics committee (approval number 2021-2414). Based on the nature of the data collected, the need for individual patient consent was waived.

### 2.1. Selection of Patients

The inclusion criteria were histologically proven diagnosis of high-grade serous ovarian carcinoma, synchronous PM, radiological FIGO stage IIIC or IV, CRS (CC-0, no visible residual tumor, or CC-1, residual disease < 2.5 mm), combined interval CRS with carboplatin-based HIPEC, and complete data available. Patients with recurrent disease, incomplete CRS, non-high-grade serous histology, early-stage disease, absence of carboplatin-based HIPEC, and incomplete data were excluded. Patients with stage IV disease who were included had a complete response to NACT at distant sites.

### 2.2. Surgical Protocol

Prior to surgery, patients received at least 3 cycles of NACT (carboplatin-paclitaxel). Radiologic evaluation confirmed the response to neoadjuvant treatment and the feasibility of complete CRS. Adjuvant chemotherapy was completed by providing an additional 3 cycles of carboplatin-paclitaxel unless individual patient factors dictated otherwise.

Surgical procedures were performed by a gynecologic oncologist and a surgical oncologist who specializes in peritoneal surface oncology. First, CRS was performed by removing all visible disease, by resecting involved organs as well as all peritoneal implants. Standard peritonectomy techniques and supracolic omentectomy were performed. When applicable, bowel resection was performed en bloc and anastomoses were completed prior to HIPEC. Pelvic and para-aortic lymphadenectomy were reserved for patients with macroscopic disease.

Postoperative evolution was followed by both teams of surgeons during the hospital stay. Patients had a peripherally inserted central catheter (PICC) to provide parenteral nutrition and a nasogastric tube in place until resuming passage of gas. A diet for the patient was then initiated and slowly advanced.

### 2.3. Hyperthermic Intraperitoneal Chemotherapy (HIPEC)

A closed-HIPEC technique was performed with carboplatin at a target temperature of 42 °C. The HIPEC regimen was modified during the study period. Patients were originally given a fixed dose of 800 to 900 mg for 60 min until 2017, when the protocol was amended to use an area under the curve (AUC) of 8 to 10, averaging a dose of 1000 mg for 90 min. The agent was diluted in 500 mL of normal saline and infused at a constant rate.

### 2.4. Postoperative Morbidity

Adverse events were reviewed in patients’ charts during admission and up to 90 days in the postoperative period. They were classified based on the Clavien–Dindo classification of surgical complications [19]. Grade I and II complications were addressed medically or by observation; serious complications, classified as grade III–IV, required either interventional or surgical procedures or intensive care unit admission. Perioperative death was defined as mortality within 30 days of the surgery.

### 2.5. Statistical Analysis

Data were collected from the patients’ medical charts up to February 2023. Descriptive analyses of patient characteristics as well as postoperative morbidity and mortality were conducted. Frequencies and percentages were used for ordinal and categorical variables. Continuous variables were presented using median and range. Kaplan–Meier curves were used to estimate DFS and OS. The impact of clinically relevant pre-defined variables on DFS and OS was evaluated using Cox univariate and multivariate regression models. Variables with *p* < 0.2 in the univariate analysis were included in the multivariate regression model. In accordance with the journal’s guidelines, we will provide our data for the reproducibility of this study in other centers upon request.

## 3. Results

### 3.1. Patient Characteristics

Forty patients were included in this study between July 2014 and March 2020. At diagnosis, the median age was 61 years. All patients had primary disease and a high-grade serous histology. Thirty-five patients (88%) presented with stage IIIC disease and five (12%) patients with stage IV. Median CA125 level was 856 units/mL, with ascites being present in 78% of cases. Most patients had an ECOG performance status of one and were not carriers of BRCA mutation (Table 1).

### 3.2. Treatment Characteristics

All patients received at least three cycles of NACT. Carboplatin-based HIPEC was administered in all patients, either over 60 min (25% of patients) or over 90 min (75% of patients). At the time of surgery, median peritoneal cancer index (PCI) was 13 (5–34) and CC-0 was achieved in 95% of patients [20]. Bowel resection was performed in half of the patients, of whom 80% had a rectal anastomosis and 20% required an ileostomy. No patient required a colostomy. Splenectomy was performed in six (15%) patients and partial gastrectomy in two patients (5%). Overall, 87% of patients completed three cycles of adjuvant chemotherapy, while only one patient received no adjuvant chemotherapy (Table 2).

### 3.3. Postoperative Morbidity

Median hospital stay was 13 days, with a median time of 7 days to resume a clear liquid diet. Median blood loss was 1000 mL and median procedure length was 475 min. At least one minor complication was reported in 85% of patients. Serious adverse events were seen in eight patients (20%), mainly grade IIIA and IVA. Specific descriptions of serious complications can be found in Supplementary Table S1. There were four admissions to the intensive care unit (10%), six hospital readmissions (15%) within 90 days, and no perioperative deaths (Table 3).

### 3.4. Oncological Outcomes

**Figure 1:**
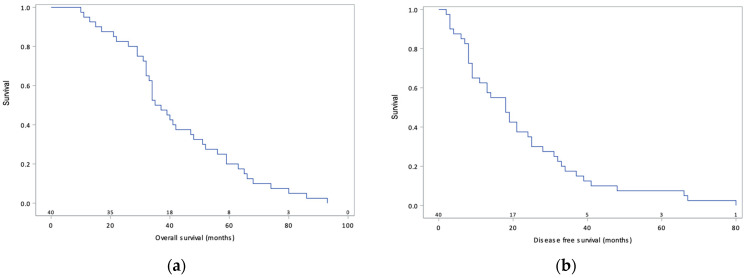
Overall survival (**a**) and disease-free survival (**b**) curves of 40 patients with primary advanced ovarian cancer treated via cytoreductive surgery combined with carboplatin-based HIPEC.

At the time of data analysis, recurrence had occurred in 33 patients (82%), 27 of whom were platinum-sensitive, and 20 deaths were recorded. Median DFS was 18.0 months (95% confidence interval 16.4–27.8) and median OS was 36.4 months (95% confidence interval 35.9–48.7) (Figure 1). The Cox multivariate regression model showed no significant impact of PCI (HR 2.23 95% CI 0.89–5.63), occurrence of severe complications (HR 1.16 95% CI 0.46–2.93), or bowel resection (HR 1.12 95% CI 0.43–2.93) on DFS. In addition, there was no significant impact of age (HR 1.45 95% CI 0.70–3.03), PCI (HR 1.50 95% CI 0.61–3.68), completeness of cytoreduction (HR 0.24 95% CI 0.03–1.85), or bowel resection (HR 1.13 95% CI 0.44–2.92) on OS (Table 4, Table 5 and Table 6).

## 4. Discussion

The objective of this study was to report on the safety and feasibility of interval complete CRS combined with carboplatin-based HIPEC on patients with advanced primary high-grade serous ovarian cancer. We found the treatment to be associated with acceptable postoperative morbidity and oncological outcomes.

The choice of agent and regimen is of utmost importance in the treatment of advanced EOC, and it is important to understand the rationale of our local practice. CRS combined with HIPEC was initiated in 2013 at our center. At the time, there was still great debate on the utilization and benefits of HIPEC and little concern about the preferred regimen for ovarian cancer. Most of our local experience was sourced from oncologic surgeons who specialize in peritoneal metastases. The initial regimen to be used was oxaliplatin (460 mg/m^2^ for 30 min IP) in combination with 5-fluorouracil (400 mg/m^2^ IV) and leucovorin (20 mg/m^2^ IV), to reflect the growing benefits shown for carcinomatosis related to colorectal cancer. At the time, our group also participated in the OV-21 trial [21], where normothermic intraperitoneal carboplatin was used in addition to intravenous (IV) chemotherapy. The cisplatin arm was discontinued early because it failed to meet the pre-set superiority rule. The literature regarding normothermic use of intraperitoneal (IP) carboplatin was reassuring in terms of its safety and it was found to be more beneficial than standard IV chemotherapy alone. It is important to keep in mind that thiosulfate renal protection protocols were not routine with cisplatin-based HIPEC and renal toxicity was a potential concern. The exact impact of thiosulfate on the intraperitoneal and systemic levels of cisplatin remains unknown, since as a chelator, it could potentially decrease its levels. The decision to go forward was also based on abstracts presented at conferences and multiple discussions with international experts in the field. Therefore, although supporting evidence was scarce, the protocol instituted was carboplatin-based HIPEC at 800–900 mg for 60 min. Maximal doses were conservative to limit toxicities.

As previously mentioned, the HIPEC regimen was modified during the study period, which can hinder the interpretation of the data. The doses and length of carboplatin used for HIPEC have been increased and our current protocol is to use carboplatin AUC 10 for 90 min. The rationale for the increase in both dose and duration was to better reflect the evolving literature on cisplatin- and carboplatin-based HIPEC [7,16]. In June 2017, Mendevil et al. published their experience with this new regimen that was performed for 90 min. They presented a cohort with reasonable toxicity who had no grade three or four complications. They showed a decreased risk of progression as well as a benefit for DFS through the use of CRS combined with carboplatin-based HIPEC. Based on their findings, we adjusted the dose at our center to be able to compare our cohort to the growing literature on the subject.

To be considered for HIPEC, all patients had to have stable or regressing disease after at least three cycles of NACT, which is consistent with current NCCN guidelines [9]. In this study, achieving complete CRS resulted in similar operative time (475 min vs. 279–492 min), hospitalization time (13 days vs. 10–18 days), intraoperative blood loss, and completion of adjuvant chemotherapy compared to the available literature on cisplatin-based HIPEC [2,7,22]. As mentioned earlier, all patients had a nasogastric tube and a PICC line that was inserted postoperatively and were kept NPO until resuming passage of gas. The rationale was to prevent aspiration pneumonia, prevent vomiting, and allow for adequate tolerance of diet once initiated. An ERAS—Early Recovery After Surgery—protocol was not instituted prior to the end of this study at our center. This translates into longer time to initiate diet and may slow recovery compared to newer comprehensive recovery protocols. Operative length could be explained by the extensive surgical approach at our center, with 50% of our patients undergoing bowel resection, as compared to 24% in OVHIPEC [7].

In our study, serious adverse events occurred in 20% of patients and resulted in six readmissions to the hospital. This is consistent with the current literature, as most trials have reported rates of serious adverse events ranging from 9 to 40% [17,18]. There were no perioperative deaths. Minor complications were frequent and consisted mostly of electrolyte disorders, anemia requiring transfusion, elevated liver enzymes, mild thrombocytopenia, and ileus [23,24,25]. Postoperative acute kidney injury occurred in two patients (5%) compared to a known rate of 8 to 40% with the use of cisplatin-based HIPEC.

Median DFS was 18.0 months, a duration consistent with the results of the OVHIPEC trial [7], which demonstrated a DFS of 14.2 months. Although the cohort in the present study is smaller, carboplatin-based HIPEC appears to provide similar short-term oncological outcomes compared to cisplatin. These results are also consistent with two recent phase II studies that used carboplatin-based HIPEC, where DFS was 11.2 months in a study by Menzies et al. [18] and 12.3 months in a study by Zivanovic et al. [26]. Median OS was 36.4 months, which is lower than the 45.7 months reported by Van Driel [7]. The seemingly lower survival observed in our cohort is attributable to a shorter follow-up period (3.0 years vs. 4.7 years), considering that only 20 patients (50% of the cohort) were followed for at least 36 months. While our cohort was only composed of patients with residual disease < 2.5 mm, patients with stage IV disease were included, which could have affected prognosis. In the 2018 landmark study [7], it is important to acknowledge that significantly more patients in the surgery-only group had platinum-resistant disease (40%) compared to 26% in the surgery and HIPEC group [8], which, given its aggressive nature, worsened the prognosis of the former group.

With recent growing interest and evidence regarding carboplatin-based HIPEC, this study is the most uniform and second largest cohort evaluating primary advanced high-grade serous ovarian cancer patients undergoing primary interval CRS and HIPEC. Smaller studies have included patients in the primary, interval, or secondary cytoreductive surgery, and all epithelial histologies were included [11,14,15,16]. Singh et al. [17] published a study with the largest cohort treated with carboplatin-based HIPEC, comprising 89 patients with advanced EOC, where 86% underwent IDS with HIPEC. Mendevil et al. [16] published a study with a cohort of 69 patients who were mostly undergoing upfront CRS with carboplatin-based HIPEC and reported a 3-year survival rate of 82.6% in the HIPEC group compared to 50% in our cohort. Unsurprisingly, they reported no grade three or four hematological complications, which is consistent with our results showing that most severe complications were infectious (Supplementary Table S1). One could presume that because HIPEC is principally localized and extracted thereafter, the toxicity is attenuated, facilitating patient tolerability. Mikkelsen’s study with a 25-patient cohort [14] demonstrated a 44% risk of severe complications and 25% readmission in upfront or interval CRS with carboplatin-based HIPEC. The higher complication rate could be attributable to more extensive surgery to achieve CC-0, considering that 56% of their patients had FIGO stage IV EOC at diagnosis.

We did not identify risk factors for recurrence; none of the pre-specified variables (i.e., age, PCI, CC, severe complication, and bowel resection) significantly affected DFS and OS. The occurrence of severe postoperative complications and bowel resections did not worsen the oncological outcomes, which reiterates the importance of maximal surgical efforts to reach complete CRS prior to HIPEC in patients when it is safe to do so. Although it is confirmed that completeness of cytoreduction is the most important prognostic factor [4], CC was not statistically significant in our cohort. This lack of significance most likely stems from the small number of patients with CC-1 in the cohort, only two patients. A trend favoring CC-0 is seen when interpreting the multivariate regression model for OS, but no specific conclusion can be drawn. In this study, all procedures were performed by the same team of gynecologic oncologists and surgical oncologists. Their systematic approach and focus on performing complete CRS can explain the high rate of complete CRS achieved, which often included organ resection and complex peritonectomy techniques [2,7,14]. Farrell’s cohort showed similar outcomes when reporting an interdisciplinary approach in CRS at their Australian center, achieving complete CC in 98% of patients [27].

### 4.1. Strengths and Weaknesses

One of the strengths of this study is the homogeneity of the cohort, which prevents the confounding effects of different histologies and treatment approaches (primary versus interval versus secondary CRS). However, these findings must be interpreted in light of several limitations, including the retrospective cohort design and the surgical and adjuvant treatment decisions determined by a single center. As we aimed to maintain the homogeneity of the cohort, the resulting smaller sample size limited data interpretation. The relatively short follow-up period and inclusion of patients with stage IV disease may also have affected the DFS and OS rates. Some studies have shown similar results for stage IV patients following interval CRS with HIPEC [28,29]. Although statistical significance was not observed consistently, results for stage IV patients show a tendency for shorter DFS and OS.

### 4.2. Impact for Future Research

Small retrospective studies, however thought-provoking, are underpowered to provide convincing evidence for the survival benefits of alternative options. Long-term prognostic outcomes of carboplatin-based HIPEC should first be evaluated further and then put into a broader perspective of the drawbacks within the HIPEC literature. Benefits for specific populations, such as patients presenting previous toxicities to cisplatin, chronic renal disease, or allergy to cisplatin, for example, should be assessed in an effort to minimize morbidity.

Eventually, to be considered as a possible alternative regimen, carboplatin-based HIPEC needs to be formally compared in a prospective randomized controlled trial to cisplatin-based HIPEC, the current standard of care. We believe there is a need to deepen the knowledge surrounding carboplatin-based HIPEC in an effort to minimize morbidity and offer the best possible oncological outcomes.

## 5. Conclusions

We propose that complete interval CRS combined with carboplatin-based HIPEC for advanced primary high-grade serous ovarian cancer is feasible, as it produced acceptable morbidity and oncological outcomes in our cohort when compared to other studies using cisplatin-based HIPEC. Larger prospective studies are required to determine long-term oncological outcomes on a larger scale.

## Figures and Tables

**Table 1:** Patient characteristics.

	No. of Patients (*n* = 40)
Median age at diagnosis (range)—years	61 (43–73)
Median age at HIPEC (range)—years	61 (44–73)
Median age at death (range)—years	68 (54–75)
Median BMI (range)	25.0 (17.7–46.0)
Primary disease—no. (%)	40 (100)
ECOG performance status—no. (%)	
0	7 (18)
1	32 (80)
2	1 (2)
BRCA status—no. (%)	
BRCA1	2 (5)
BRCA2	3 (7)
Negative	27 (68)
Unknown	8 (20)
Histology—no. (%)	
High grade serous	40 (100)
Stage—no. (%)	
IIIC	35 (88)
IVA	2 (5)
IVB	3 (7)
Median Ca125 level (range)—units/mL	856 (22 → 20,000)
Imaging—no. (%)	
Presence of ascites	31 (78)

**Table 2:** Treatment characteristics.

	No. of Patients (*n* = 40)
Neoadjuvant chemotherapy	
Number of cycles—no. (%)	
0	0 (0)
3	13 (33)
4	22 (55)
5 or more	5 (12)
Cytoreductive surgery	
HIPEC agent—no. (%)	
Carboplatin	40 (100)
HIPEC duration—no. (%)	
Carboplatin 60 min	10 (25)
Carboplatin 90 min	30 (75)
Complete cytoreduction1—no. (%)	
CC-0	38 (95)
CC-1	2 (5)
Median peritoneal cancer index (range)	13 (5–34)
Peritoneal cancer index—no. (%)	
0–15	25 (63)
16 or more	15 (37)
Median blood loss (range)—mL	1000 (300–4000)
Median duration of procedure (range)—min	475 (300–900)
Supracolic omentectomy	31 (78)
Peritonectomy	26 (65)
Partial hepatectomy	2 (5)
Splenectomy	6 (15)
Bowel resection—no. (%)	20 (50)
Small bowel resection	2 (5)
Colon resection	20 (50)
Partial gastrectomy	2 (5)
Bowel resection with ileostomy	4 (10)
Bowel resection with colostomy	0 (0)
Rectal anastomosis	16 (40)
Paraaortic lymph node dissection	10 (25)
Adjuvant chemotherapy	
Number of cycles—no. (%)	
0	1 (3)
1–2	4 (10)
3	35 (87)
Total number of cycles—no. (%)	
3–5	2 (5)
6–7	33 (83)
8 or more	5 (12)

CC-0, no visible residual tumor; CC-1, residual disease < 2.5 mm.

**Table 3:** Surgical outcomes and postoperative morbidity.

	No. of Patients (*n* = 40)
Median blood loss (range)—mL	1000 (300–4000)
Median duration of procedure (range)—min	475 (300–900)
Median Ca125 levels post-chemotherapy (range)—kU/L	15.4 (3.9–142.4)
Median length of hospital stay (range)—d	13 (8–80)
Median time with nasogastric tube (range)—d	6 (0–13)
Median time for passage of gas—d	5 (3–10)
Median time to initiate diet (range)—d	7 (2–13)
Median duration of parenteral nutrition (range)—d	10 (0–49)
Intensive care unit admission—no. (%)	4 (10)
Readmission < 90 days—no. (%)	6 (15)
Blood transfusion—no. (%)	
Perioperative transfusion	16 (40)
Postoperative transfusion	17 (43)
Perioperative death—no. (%)	0 (0)
Surgical morbidity as per Clavien–Dindo classification	
Minor complications—no. (%)	34 (85)
Grade 1	31 (78)
Grade 2	21 (53)
Major complications—no. (%)	8 (20)
Grade 3A	3 (8)
Grade 3B	2 (5)
Grade 4A	4 (10)
Grade 4B	1 (3)
Grade 5 complication—no. (%)	0 (0)

**Table 4:** Oncological outcomes.

	No. of Patients (*n* = 40)
Median disease-free survival (95% confidence interval)—mo	18.0 (16.4–27.8)
Median overall survival (95% confidence interval)—mo	36.4 (35.9–48.7)
Recurrence interval following CC-0—no. (%)	
Platinum-resistant (<6 months)	5 (13)
Platinum-sensitive (>6 months)	26 (69)
No recurrence	7 (18)
Progression interval following CC-1—no. (%)	
Platinum-resistant (<6 months)	1 (50)
Platinum-sensitive (>6 months)	1 (50)

**Table 5:** Cox PH regression of prognostic factors of DFS.

Variables	Univariate HR (95% CI)	*p* Value	Multivariate HR (95% CI)	*p* Value
Age < 60	-	-	-	-
Age ≥ 60	1.07 (0.57–2.02)	0.83	NA	
PCI 0–15	-	-	-	-
PCI ≥ 16	2.53 (1.26–4.96)	0.01	2.23 (0.89–5.63)	0.09
CC-0	-	-	-	-
CC-1	2.30 (0.54–9.83)	0.26	NA	
No severe complication	-	-	-	-
Occurrence of severe complications	1.70 (0.76–3.79)	0.19	1.16 (0.46–2.93)	0.75
No bowel resection	-	-	-	-
Bowel resection	1.89 (0.99–3.62)	0.05	1.12 (0.43–2.93)	0.82

**Table 6:** Cox PH regression of prognostic factors of OS.

Variables	Univariate HR (95% CI)	*p* Value	Multivariate HR (95% CI)	*p* Value
Age < 60	-	-	-	-
Age ≥ 60	1.79 (0.93–3.45)	0.08	1.46 (0.70–3.03)	0.32
PCI 0–15	-	-	-	-
PCI ≥ 16	1.87 (0.96–3.64)	0.07	1.50 (0.61–3.68)	0.38
CC-0	-	-	-	-
CC-1	0.22 (0.03–1.65)	0.14	0.24 (0.03–1.85)	0.17
No severe complication	-	-	-	-
Occurrence of severe complications	1.33 (0.60–2.96)	0.48	NA	
No bowel resection	-	-	-	-
Bowel resection	1.80 (0.94–3.46)	0.08	1.13 (0.44–2.92)	0.80

## Data Availability

The data presented in this study are available on request from the corresponding author. The data are not publicly available due to ongoing projects in similar areas.

## Supplementary Materials

The following supporting information can be downloaded at: https://www.mdpi.com/article/10.3390/curroncol30120748/s1, Table S1: Description of severe adverse events.
