# Supplementary material for: Combined Interval Cytoreductive Surgery and Carboplatin-Based Hyperthermic Intraperitoneal Chemotherapy in Advanced Primary High-Grade Serous Ovarian Cancer
Source: Curr Oncol. 2023 Dec 1;30(12):10272–82. doi: 10.3390/curroncol30120748 (PMC10742627; doi:10.3390/curroncol30120748)
Supplement: Supplementary file 1 [file curroncol-30-00748-s001.zip › curroncol-2687378-supplementary.pdf]

Current Oncology

**Supplementary Table S1.** Description of severe adverse events

| | |
|---|---|
| Patient 1 | Readmisison to ICU 30 days post-op for hypovolemia and acute kidney injury |
| Patient 2 | Extubation failure due to glottis edema causing asystole secondary to respiratory insufficiency and requiring ICU admission (length of surgery 510 minutes) |
| Patient 3 | Septic shock requiring ICU admission |
| Patient 4 | Septic shock required ICU admission |
| Patient 5 | Opioid overdose requiring ICU admission |
| Patient 6 | Readmission for drainage of hepatic abscess |
| Patient 7 | Readmission for small bowel obstruction requiring surgery for adhesion lysis |
| Patient 8 | Readmission for bacterial peritonitis requiring ascites drainage |
